# The association between Hepcidin and arterial stiffness in a community-dwelling population

**DOI:** 10.1186/s12944-018-0866-6

**Published:** 2018-10-30

**Authors:** Xiaona Wang, Li Sheng, Ping Ye, Ruihua Cao, Xu Yang, Wenkai Xiao, Yun Zhang, Yongyi Bai, Hongmei Wu

**Affiliations:** Department of Geriatric Cardiology, Chinese PLA General Hospital, Fuxing Road #28, Beijing, 100853 China

**Keywords:** Hepcidin, HDL3-C, Carotid–femoral pulse wave velocity

## Abstract

**Background:**

An association of hepcidin with cardiovascular (CV) disease and atherosclerosis has been reported in different patient groups. However, it has not been well described clinically the association between hepcidin and arterial stiffness. In this study,We analysed the possible mechanism of Hepcidin and arterial stiffness.

**Methods:**

This article related measurements of plasma hepcidin and arterial stiffness (carotid–femoral pulse wave velocity [PWV]) in a community-based sample.

**Results:**

After a median follow-up interval of 4.8 years, multiple linear regression analysis revealed that hepcidin was independently associated with carotid–femoral PWV (β = 1.498, *P* < 0.001). In a multivariable linear regression analysis, HDL3-C levels were negatively and independently associated with hepcidin at baseline (β = − 0.857, *P* = 0.024). HDL2-C was not associated with hepcidin at baseline (β = − 1.121, *P* = 0.133).

**Conclusions:**

We found an association between baseline hepcidin and follow-up arterial stiffness that was independent of age, gender and other vascular risk factors. We also identified an association between hepcidin and HDL3-C at baseline, which indicates that the HDL3-C level may reflect the change in cholesterol efflux from peripheral arteries and partly explain the relationship between hepcidin and the change of arterial stiffness.

## Background

An abnormality in iron metabolism has been recognized as a strong predictor of cardiovascular disease [1, 2]. The Kuopio Ischaemic Heart Disease Risk Factor Study including 1931 Finnish men with asymptomatic for heart disease confirmed that elevated stored iron levels increase the incidence of coronary heart disease [3]. Meanwhile, the Bruneck Research found that the incidence of atherogenesis in male patients is three times that of premenopausal women which demonstrates an a strong correlation between high levels iron and atherogenesis [4]. Hepcidin was first discovered as a peptide with antimicrobial properties in 2001 [5, 6]. Recently, hepcidin has been recognized as the key hormone in the regulation of iron balance and iron recycling [7, 8]. Some studies have reported a strong association between hepcidin and cardiovascular (CV) disease and atherosclerosis in different patient groups [9–11]. However, mechanistic studies have mainly focused on hepcidin-mediated iron metabolism.

Recently, the pharmacologic suppression of hepcidin has been shown to increase the expression of ABCA1 and ABCG1 and lipid efflux via the macrophage-specific expression of cholesterol efflux transporters and to reduce foam cell formation and atherosclerosis in vivo and vitro [12]. Reverse cholesterol transport (RCT) is the main process of HDL that opposes atherosclerosis. Great emphasis has been placed on the role of individual HDL subclasses (HDL2-C, HDL3-C), which are not equally atheroprotective [13]. Therefore, the attention on HDL-C has now started to transfer away from the cholesterol-centric theory towards alternative indices of HDL such as subclass distribution, particle size, and measures of HDL functionality. However, what is lacking is a clinical study on hepcidin and HDL subclasses.

Carotid-femoral PWV has been generally regarded as a “gold standard” indicator of subclinical vascular disease as well as cardiovascular mortality in many epidemiological studies [14]. In this article, we puts forward the relationship between hepcidin and arterial stiffness by studying: (1) the predictive connection of baseline hepcidin values with follow-up arterial stiffness in a large longitudinal sample in Beijing; and (2) the relationship between HDL subclasses with hepcidin at baseline to explain a possible clinical mechanism.

## Methods

### Subjects

The population in this study included 1447 subjects who lived in Shijingshan community Pingguoyuan area of Beijing, China. The original data was from a routine health check-up of 1680 subjects between September 2007 and January 2009. We conducted the first follow-up evaluation to collect information from these subjects prospectively from February 1 to September 30, 2013, and 1499 subjects were successfully evaluated (181 subjects were lost, follow-up rate was 89.2%). The median follow-up interval for the original 1499 subjects was 4.8 years. The final number of subjects is 1447; 52 samples were excluded (death). No differences other than in baseline risk factors were noted in participants who completed baseline and follow-up assessments.

### Clinical data collection

Clinical data were collected via a face-to-face questionnaire survey from all subjects to ascertain new CVD events during these visits. Data were categorized as below:

- Data about prevalent diseases, medical histories, lifestyle factors and family history;
- Data about urine and fasting blood samples;
- Data about blood pressure and other anthropometric measurements (obtained by trained physicians).

### Biomarker variable determination

Blood samples were collected in centrifuge tubes after subjects fasted overnight and were centrifuged them for 15 min at 1200×g. Serum aliquots were placed at − 80 °C before use. Hepcidin concentrations were measured using a commercially available quantitative sandwich ELISA assay (CY-8079; CycLex Co., Nagano, Japan). HDL 2 and HDL 3 were separated in a Himac centrifuge with a PR80A rotor (Hitachi, Tokyo, Japan) [15]. Concentrations of fasting blood glucose (FBG), high-density lipoprotein cholesterol (HDL-C), low-density cholesterol cholesterol (LDL-C), triglyceride (TG) and total cholesterol (TC) were evaluated on a Roche autoanalyzer (Roche Diagnostics, Indianapolis, IN, USA). Concentrations of serum creatinine were measured on Hitachi 7600 autoanalyser (Hitachi, Tokyo, Japan). All testing was performed in the same laboratory following the criteria of the World Health Organization Lipid Reference Laboratories.

### Assessment of arterial stiffness

Before the experiment, everyone avoided smoking, drinking, tea and caffeine. Arterial stiffness was measured and calculated by carotid–femoral pulse wave velocity (cf-PWV) using Complior SP device (Createch Industrie, Massy, France). We positioned two pressure-sensitive transducers (Fukuda Denshi Co., Tokyo, Japan) over the common carotid artery and the femoral artery. Arterial stiffness was calculated according to the following formula: cf-PWV (m/s) = distance (m)/transit time (s) [16].

### Definition of variables

- Body mass index (BMI) = weight (kg) / height^2^ (m^2^).
- The estimated glomerular filtration rate (eGFR) was calculated via the below Chronic Kidney Disease Epidemiology Collaboration equation: eGFR = 141 × min (Scr/κ,1)α × max (Scr/κ, 1)-1.209 × 0.993Age × 1.018 [if female] × 1.159 [if black], where Scr is plasma creatinine (mg/dL), κ is 0.7 for females and 0.9 for males, α is − 0.329 for females and − 0.411 for males, min indicates the minimum of Scr/κ or 1, and max indicates the maximum of Scr/κ or 1.

### Statistical analyses

Median (interquartile range) or mean ± standard deviation (SD) was used to expressed baseline continuous variables were expressed as the. Percentages were used to expressed dichotomous variables.

Two categories were classified according to baseline distribution of Carotid-femoral PWV:

1. elevated levels (≥12 m/s);
2. normal level (< 12 m/s) [17].

We used t-test to describe baseline continuous variables and chi-square to describe baseline categorical variables between elevated and normal carotid-femoral PWV groups.

Pearson’s correlation and multiple linear regression analysis were used to describe the correlations between the baseline hepcidin level and follow-up carotid-femoral PWV. Regression models were adjusted for age and gender as well as hypertension, SBP, DBP, Diabetes (DM), smoking, BMI, levels of glucose, TC, HDL-C, TG, LDL-C and eGFR. In addition, we investigated the association of hepcidin in combination with HDL subclasses (HDL2-C, HDL3-C) at baseline by leveraging Pearson’s correlation and multiple linear regression analysis.

We used receiver operating characteristic (ROC) curves to assess the ability of the baseline hepcidin level indices to predict carotid-femoral PWV.

We used SPSS software for Windows, version 13.0 (SPSS, Chicago, IL, USA). *P*-values of < 0.05 were considered statistically significant.

## Results

### Baseline parameters in elevated and normal carotid-femoral PWV groups

Characteristics of all 1447 subjects without cardiovascular disease at baseline according to baseline carotid-femoral PWV are summarized in Table 1. The mean age (±SD) of the population in the study was 61.30 ± 11.4 years. Age, levels of SBP, FBG, TG, LDL-C and hepcidin, as well as rates of male gender and smoking were significantly higher, but eGFR and HDL3-C levels were lower in the elevated carotid-femoral PWV group compared with normal cfPWV group.

**Table 1:** Baseline characteristics of the subjects

Variables	All subjects (*n* = 1447)	Carotid-femoral PWV ≥ 12 (*n* = 571)	Carotid-femoral PWV < 12 (*n* = 876)	*P*-value
Age	61.30 ± 11.4	65.59 ± 9.03	54.14 ± 10.11	< 0.001
Male (%)	601 (41.53)	270 (47.28)	331 (37.78)	< 0.001
BMI	25.41 ± 3.32	25.41 ± 3.28	25.26 ± 4.17	0.583
SBP(mmHg)	128.7 ± 17.7	135.38 ± 18.37	124.37 ± 15.93	< 0.001
DBP(mmHg)	77.11 ± 10.26	77.00 ± 10.92	77.18 ± 9.82	0.762
FBG (mmol/l)	5.39 ± 1.65	5.63 ± 1.87	5.21 ± 1.35	< 0.001
TG(mmol/l)	1.80 ± 1.24	1.92 ± 1.27	1.74 ± 1.24	0.017
TC (mmol/l)	5.01 ± 0.93	5.02 ± 0.97	5.01 ± 0.89	0.845
HDL-C(mmol/l)	50.83 ± 13.02	50.32 ± 12.60	51.47 ± 13.33	0.191
HDL2-C	29.85 ± 9.13	29.69 ± 9.44	30.05 ± 9.08	0.593
HDL3-C	20.98 ± 4.22	20.62 ± 4.12	21.42 ± 4.38	0.006
LDL-C(mmol/l)	2.91 ± 0.71	2.96 ± 0.73	2.87 ± 0.69	0.030
eGFR (ml/min)	94.2 ± 14.30	88.18 ± 14.19	98.63 ± 12.45	< 0.001
Hepcidin	226.43 ± 45.53	209.94 ± 43.34	227.20 ± 48.83	< 0.001
Smokers	380 (26.26)	175 (30.65)	205 (23.40)	< 0.001
Hypertension	755 (52.17)	418 (73.20)	337 (38.47)	< 0.001

*BMI* body mass index, *SBP* systolic blood, *DBP* diastolic blood pressure, *FBG* fast blood glucose, *TG* triglyceride, *TC* total cholesterol, *HDL-C* high- density lipoprotein cholesterol, *LDL-C* low-density lipoprotein cholesterol, *eGFR* estimated glome-rular filtration rate, *CHD* coronary heart disease, *PWV* pulse-wave velocity

### Association of baseline hepcidin with follow-up carotid-femoral PWV

**Fig. 1:**
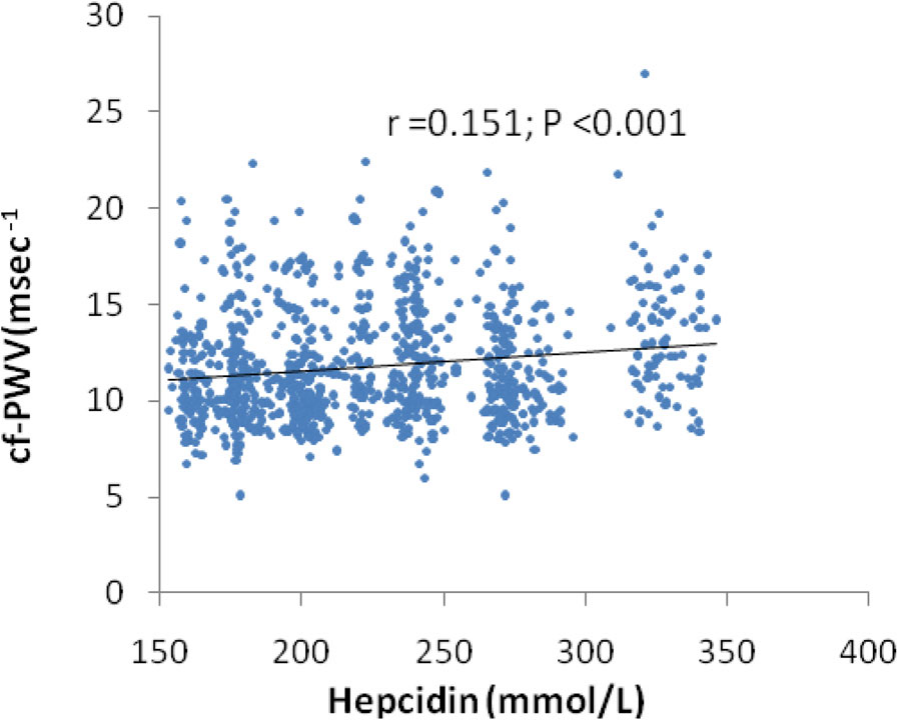
Association of baseline hepcidin with follow-up Carotid-femoral PWV

Age (*r* = 0.538; *P* < 0.001), hepcidin (*r* = 0.151; *P* < 0.001, Fig. 1), TG (*r* = 0.747; *P* < 0.001), SBP (*r* = 0.228; *P* < 0.001) and FBG (*r* = 0.118; *P* < 0.001) were related to carotid-femoral PWV significantly and positively. eGFR (*r* = − 0.376; P < 0.001) has a inversely relationship with carotid-femoral PWV.

Table 2 showed the relationship between baseline hepcidin and follow-up arterial stiffness in multivariable linear regression analysis. Follow-up carotid-femoral PWV had a positively and independently association with baseline hepcidin level (β = 1.498, P < 0.001). Furthermore, male gender, hypertension, TG, LDL-C, TC, SBP, BMI and fasting blood glucose levels were positively and independently associated with carotid-femoral PWV whereas eGFR was negatively associated with follow-up carotid-femoral PWV.

**Table 2:** Multiple linear regression analysis of baseline parameters and follow-up arterial stiffness

	carotid–femoral PWV		carotid–femoral PWV		
	*r*	*P*-value	β	CI	*P*-value
All subjects(*n* = 1447)					
Age	0.538	< 0.001	−0.025	0.012~ 0.037	< 0.001
Male	–	–	0.725	0.340~ 1.110	< 0.001
Smoking	–	–	0.320	− 0.086~ 0.725	0.122
Diabetes	–	–	0.232	0.058~ 0.522	0.117
Hypertension	–	–	0.883	0.501~ 1.265	< 0.001
Hepcidin	0.151	< 0.001	1.498	0.696~ 2.300	< 0.001
TG^a^	0.747	< 0.001	0.367	0.140~ 0.593	0.002
HDL-C^a^	0.065	0.049	0.192	− 0.382~ 0.767	0.511
LDL-C	0.014	0.679	0.741	0.240~ 1.242	< 0.004
TC	0.049	0.132	0.549	0.153~ 0.945	0.007
SBP	0.228	< 0.001	0.046	0.033~ 0.060	< 0.001
DBP	−0.038	0.252	0.059	− 0.079~ − 0.040	< 0.001
BMI	0.054	0.098	0.090	0.037~ 0.144	< 0.001
FBG	0.118	< 0.001	0.293	0.187~ 0.398	< 0.001
eGFR^a^	− 0.376	0.001	− 0.059	− 0.072~ − 0.047	< 0.001

*TC* total cholesterol, *HDL-C* high- density lipoprotein cholesterol, *TG* triglyceride, *LDL-C* low-density lipoprotein cholesterol, *SBP* systolic blood pressure, *DBP* diastolic blood pressure, *BMI* body mass index, *FBG* fast blood glucose, *eGFR* estimated glomerular filtration rate, *PWV* pulse wave velocity

^a^: natural logarithm transformed

§: Covariates in the multiple-adjusted models included age, gender, hypertension, DM, current smoking, levels of plasma TC, TG, LDL-C, HDL-C, SBP, DBP,FBG, BMI and eGFR

**Fig. 2:**
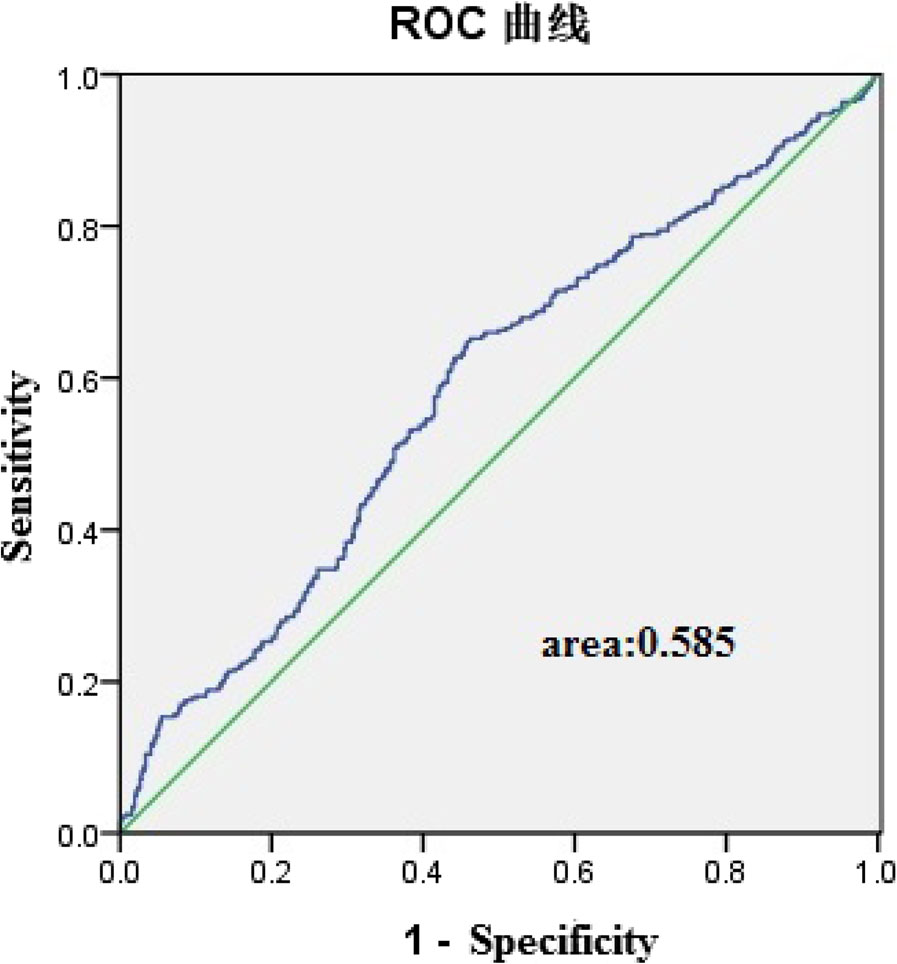
The receiver operating characteristic (ROC) curves for the assessment of the hepcidin level indices as predictors of carotid-femoral PWV

ROC curves for the assessment of the hepcidin level indices as predictors of carotid-femoral PWV are presented in Fig. 2.

### Association of hepcidin with HDL subclasses (HDL2-C, HDL3-C)

**Fig. 3:**
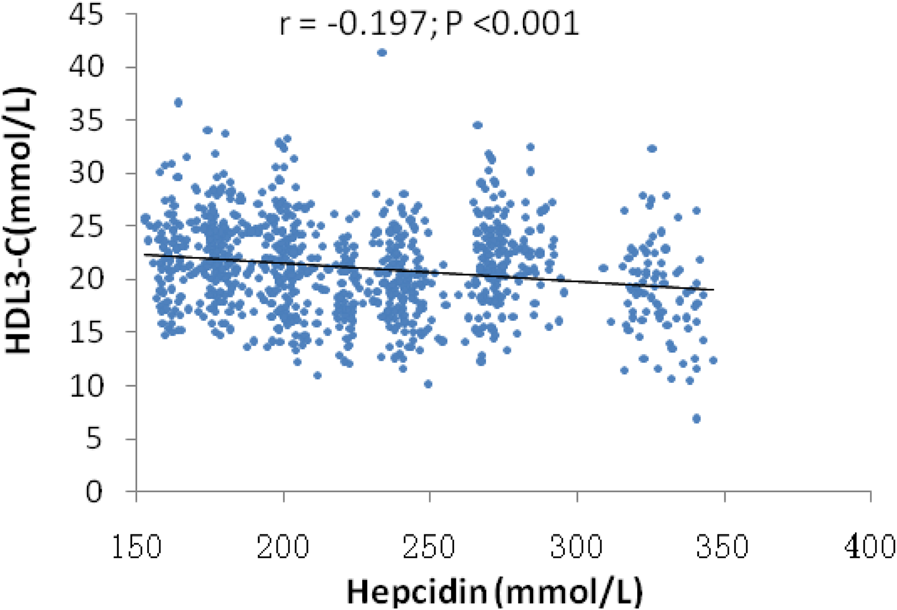
Hepcidin was significantly and negatively related to HDL3-C

The associations between HDL subclasses as a continuous variable (natural logarithm transformed) and hepcidin at baseline are summarized in Table 3. Hepcidin was significantly and negatively related to HDL3-C (*r* = − 0.197; *P* < 0.001, Fig. 3). In multivariable linear regression analysis, HDL3-C levels were negatively and independently associated with hepcidin at baseline (β = − 0.857, *P* = 0.024). HDL2-C was not associated with hepcidin at baseline (β = − 1.121, *P* = 0.133).

**Table 3:** Association of hepcidin with HDL subclasses (HDL2-C, HDL3-C)

	HDL2-C			HDL3-C		
	β	CI	*P*-value	β	CI	*P*-value
All subjects(*n* = 1447)						
hepc	−1.121	0.133~ − 2.584	0.133	−0.857	− 1.599~ − 0.114	0.024
Male	−4.243	−5.352~ − 3.131	< 0.001	− 0.602	− 1.165~ − 0.039	0.036
age	0.133	0.078~ 0.188	< 0.001	0.033	0.005~ 0.061	0.021
smoking	−0.001	0.171~ 1.076	0.007	−0.220	− 0.835~ 0.395	0.482
BMI	−0.098	−0.188~ − 0.008	0.032	0.001	−0.047~ 0.045	0.960
TC	9.042	7.993~ 10.090	< 0.001	2.894	2.362~ 3.426	< 0.001
LDL-C	−0.892	−10.097~ − 7.358	0.002	−1.450	−2.146~ − 0.755	< 0.001
TG^a^	−9.835	−10.840~ − 8.810	< 0.001	−1.688	− 2.203~ − 1.172	< 0.001
FBG	−0.265	−0.580~ 0.050	0.099	0.044	- 0.116~ 0.204	0.589
eGFR^a^	0.060	0.016~ 0.104	0.008	0.013	−0.010~ 0.035	0.262

*TC* total cholesterol, *HDL-C* high- density lipoprotein cholesterol, *TG* triglyceride, *LDL-C* low-density lipoprotein cholesterol, *BMI* body mass index, *FBG* fast blood glucose, *eGFR* estimated glomerular filtration rate, *PWV* pulse wave velocity

^a^: natural logarithm transformed

§: Covariates in the multiple-adjusted models included age, gender, hypertension, DM, current smoking, levels of plasma TC,TG,HDL-C,LDL-C,FBG, BMI, eGFR

**Fig. 4:**
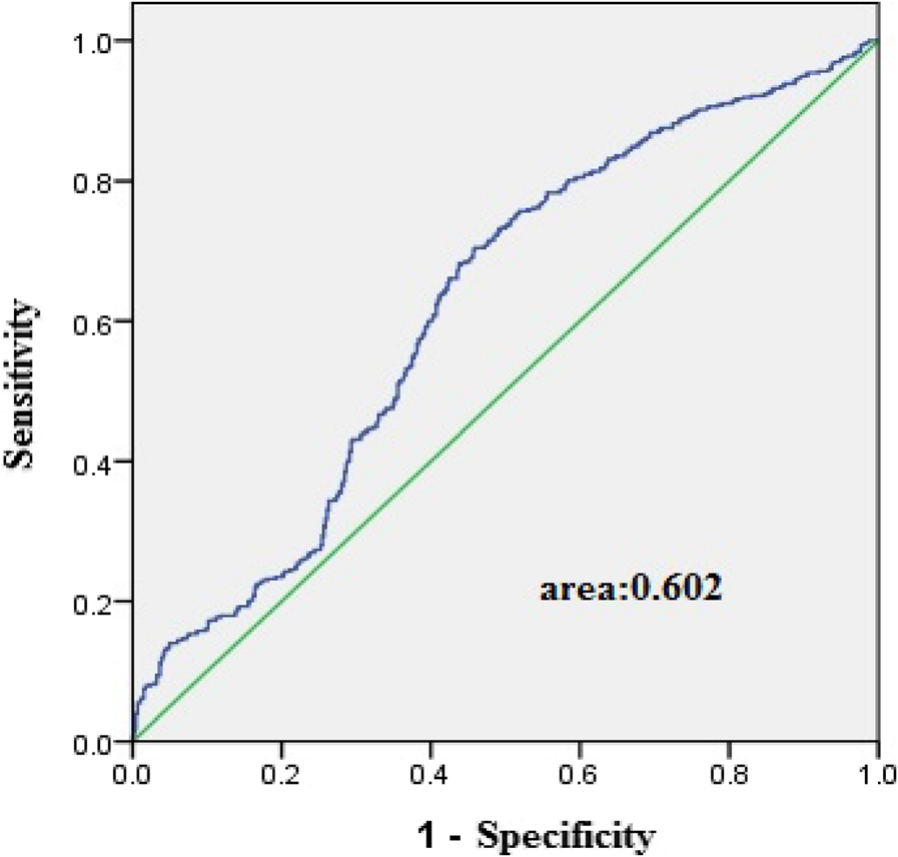
The receiver operating characteristic (ROC) curves for the assessment of the association of hepcidin and HDL3-C

ROC curves for the assessment of the association of hepcidin and HDL3-C is presented in Fig. 4.

## Discussion

Until then, no studies had been conducted to prove the relationships among hepcidin levels, HDL3-C and carotid-femoral PWV. In the present longitudinal study, we demonstrated the following: 1) An association between baseline hepcidin and follow-up arterial stiffness independent of age, gender and other vascular risk factors. 2) An association between hepcidin and HDL3-C at baseline, which indicates that hepcidin may be a promising target to increase reverse cholesterol transport from macrophages and inhibit atherosclerosis.

Sullivan proposed that hepcidin would increase the risk of atherosclerosis by increasing iron aggregation in macrophages, and subsequently increase lipid peroxidation and foam cell formation within atherosclerotic plaques [18]. A study that included 766 participants aged 46 to 67 years confirmed that hepcidin affected the development of atherosclerosis in women [19]. Valenti L et al. that increased hepcidin levels had positive association with increased aortic stiffness independently in 827 consecutive outpatients [20]. Furthermore, it is founded that hepcidin-25 was independently associated with carotid plaques in patients with nonalcoholic fatty liver disease [21].

In our study, we used carotid-femoral PWV, which reflects the presence of atherosclerosis. Recently, the risk factor that arterial stiffness play in subclinical vascular disease as well as cardiovascular mortality has been recognized [22–25]. Several studies based on community population have attached importance to the role of arterial stiffness, which can be measured by the noninvasive technique pulse wave velocity (PWV) [26, 27]. Carotid-femoral PWV is the “gold standard” for the assessment of arterial stiffness; numerous epidemiological studies indicate its predictive value for CHD [28]. In addition, our research population is older, nearly half of them are over 65. The steepest rise of transmural pressure-induced arterial wall damage occurs after the age of 60 years [29–31], increasing the risk of ill effects due to other risk factors in older subjects.

In the past, the research focused on hepcidin and abnormal iron metabolism and CV risk; our current research identified an association between hepcidin and HDL3-C at baseline, which indicates that the HDL3-C level may reflect the change in cholesterol efflux from peripheral arteries and partly explain the relationship between hepcidin and the change of arterial stiffness. Some potential mechanisms support this hypothesis that hepcidin is a biomarker of carotid-femoral PWV risk, given the role of decreasing cholesterol efflux that has been observed in vitro or in vivo. In an experimental mouse study [32], the suppression of hepcidin production in the liver decreases the formation of foam cells and atherosclerosis by reducing intracellular iron content in macrophages, which increased the efflux cholesterol capacity. Hepcidin has been showed to increase reactive oxygen species and decrease cholesterol efflux by increasing intracellular iron content, which was done with human monocytes derived from atherosclerotic plaques [33]. These findings are identical to other in vivo and in vitro studies [34], showing that the interaction of (locally produced) accumulated lipids with hepcidin and trapped iron play a key role in the proatherosclerotic activation of macrophages and accelerates plaque destabilization. Moreover, Omar Saeed et al. reported that the pharmacologic suppression of hepcidin increases the expression of ABCA1 and ABCG1 and lipid efflux via the macrophage-specific expression of cholesterol efflux transporters and reduces foam cell formation and atherosclerosis [12]. Clinically, we found that the hepcidin level was negatively associated with the HDL3-C level among subjects at baseline, indicating that hepcidin may decrease cholesterol efflux, manifested as lower HDL3-C. HDL-C can been isolated into HDL2-C (‘large, buoyant’) and HDL3-C (‘small, dense’) particles, respectively, by ultracentrifugation, and HDL particles are heterogeneous in their structure, metabolism, and biological functionality [35–37]. It has been demonstrated that protein-rich HDL3 particles are even more important to atheroprotective than cholesterol-rich HDL2 particles [38–40]. HDL 3 is the main component of HDL; the majority (75%) of HDL cholesterol resides in this subclass. Martin SS et al. reported that a low HDL3-C level, but not a low HDL2-C level, was associated with an increased risk of myocardial infarction or death in secondary prevention patients, highlighting the potential value of HDL3-C [41]. HDL3 particles play a central role in RCT, extracting cholesterol from the periphery, and maturing into HDL2 particles via progressive lipidation by lecithin: cholesteryl acyltransferase [41]. The central positioning of HDL3 in RCT and greater contribution to total HDL-C may indicate that HDL3 assumes the majority of the responsibility for HDL functions, such as RCT.

The above data proves this conclusion that the HDL3-C level may reflect the change in cholesterol efflux from peripheral arteries and partly explains the relationship of hepcidin and change in arterial stiffness, but further research is needed to explore more of these mechanisms.

## Conclusions

We found an association between baseline hepcidin and follow-up arterial stiffness that was independent of age, gender and other vascular risk factors. We also identified an association between hepcidin and HDL3-C at baseline, which indicates that the HDL3-C level may reflect the change in cholesterol efflux from peripheral arteries and partly explain the relationship between hepcidin and the change of arterial stiffness.

## Abbreviations

BMI: Body mass index
cf-pwv: Arotid–femoral pulse wave velocity
cr-pwv: Carotid–radial pulse wave velocity
DBP: Diastolic blood pressure
DM: Diabetes mellitus
eGFR: Estimated glomerular filtration rate
FBG: Fasting blood glucose
HDL-C: High-density lipoprotein cholesterol
LDL-C: Low-density lipoprotein cholesterol
SBP: Systolic blood
TC: Total cholesterol
TGs: Triglycerides
